# Inflammasome inhibition blocks cardiac glycoside cell toxicity

**DOI:** 10.1074/jbc.RA119.008330

**Published:** 2019-07-12

**Authors:** Doris L. LaRock, Jenna S. Sands, Ethan Ettouati, Marine Richard, Paul J. Bushway, Eric D. Adler, Victor Nizet, Christopher N. LaRock

**Affiliations:** ‡ Department of Pediatrics, University of California San Diego, La Jolla, California 92093; § Skaggs School of Pharmacy and Pharmaceutical Sciences, University of California San Diego, La Jolla, California 92093; ¶ Department of Microbiology and Immunology, Emory School of Medicine, Atlanta, Georgia; ‖ Department of Medicine, Emory School of Medicine, Atlanta, Georgia 30322; ** Institut Supérieur de la Santé et des Bioproduits, Angers, France 49000; ‡‡ Department of Cardiovascular Medicine, University of California San Diego, La Jolla, California 92093

**Keywords:** inflammasome, cardiomyocyte, macrophage, IL-1, cell death, caspase 1 (CASP1), cardiac glycoside

## Abstract

Chronic heart failure and cardiac arrhythmias have high morbidity and mortality, and drugs for the prevention and management of these diseases are a large part of the pharmaceutical market. Among these drugs are plant-derived cardiac glycosides, which have been used by various cultures over millennia as both medicines and poisons. We report that digoxin and related compounds activate the NLRP3 inflammasome in macrophages and cardiomyocytes at concentrations achievable during clinical use. Inflammasome activation initiates the maturation and release of the inflammatory cytokine IL-1β and the programmed cell death pathway pyroptosis in a caspase-1–dependent manner. Notably, the same fluxes of potassium and calcium cations that affect heart contraction also induce inflammasome activation in human but not murine cells. Pharmaceuticals that antagonize these fluxes, including glyburide and verapamil, also inhibit inflammasome activation by cardiac glycosides. Cardiac glycoside–induced cellular cytotoxicity and IL-1β signaling are likewise antagonized by inhibitors of the NLRP3 inflammasome or the IL-1 receptor–targeting biological agent anakinra. Our results inform on the molecular mechanism by which the inflammasome integrates the diverse signals that activate it through secondary signals like cation flux. Furthermore, this mechanism suggests a contribution of the inflammasome to the toxicity and adverse events associated with cardiac glycosides use in humans and that targeted anti-inflammatories could provide an additional adjunct therapeutic countermeasure.

## Introduction

Inflammasomes mediate recognition of exogenous pathogen-associated molecular patterns and endogenous damage-associated molecular patterns indicating infection, cell damage, or cell stress. These processes can be influenced by drugs that alter cation gradients, osmolarity, membrane integrity, and metabolism (1–4). Upon activation, an inflammasome uses the protease caspase-1 to mature and release the pro-inflammatory cytokine IL-1β and activate the cell death program of pyroptosis. IL-1β and pyroptosis are key drivers of pathology in numerous diseases; thus, the efficacy of a therapeutic agent might be compromised when it activates inflammasomes.

Cardiac glycosides alter cation homeostasis in mammalian cells by inhibiting Na,K-ATPase function. Heart muscle is acutely sensitive to this action, and the consequent cellular calcium increase reduces the heart rate and increases the contractile force. Ingestion of wild or ornamental plants containing cardiac glycosides, including lily of the valley, foxglove, and oleander, can lead to lethal intoxication and is a common choice for intentional self-poisoning throughout the world (5). However, these compounds can also be therapeutically useful, and cardiac glycosides have been used to treat heart conditions for centuries, as documented by use of foxglove by the 18^th^-century botanist and physician William Withering (digitalis/digoxin) (6). Cardiac glycosides have a challengingly narrow therapeutic window, a prime example of Paracelsus's adage “the dose makes the poison.” Intoxication is a common and serious adverse event observed across numerous clinical trials, and a long-standing controversy has been whether their continued use is justified (7–10). Their low cost has made them particularly attractive for the management of rheumatic heart disease induced by group A *Streptococcus*, which is most prevalent in resource-poor countries, but the potential therapeutic benefit may also be associated with increased mortality (11). Cardiac glycosides remain a recommended alternative for management of heart failure by the American College of Cardiology Foundation/American Heart Association Task Force on Practice Guidelines (12).

Most often, the presentation of intoxication is cardiac dysfunction with arrhythmia or atrioventricular block, although cell toxicity by a variety of pathways has been reported for some cardiac glycosides (13–15). Here we report that digoxin and other cardiac glycosides potently activate the inflammasome in human cells, including cardiomyocytes. This sensitivity exceeds that reported previously in mice (14), mirroring the known species-specific susceptibility of the Na,K-ATPase cardiac glycosides target (16). Inflammasome activation results in maturation and release of the proinflammatory cytokine IL-1β and death of the intoxicated cell by pyroptosis. Food and Drug Administration (FDA)^3^ approved and investigational pharmaceutical compounds that inhibit the inflammasome components NLRP3 or caspase-1, and/or block IL-1β receptor signaling, prevent the activation of IL-1β and cell death observed in response to cardiac glycosides.

## Results

### Cardiac glycosides are cytotoxic

**Figure 1.**
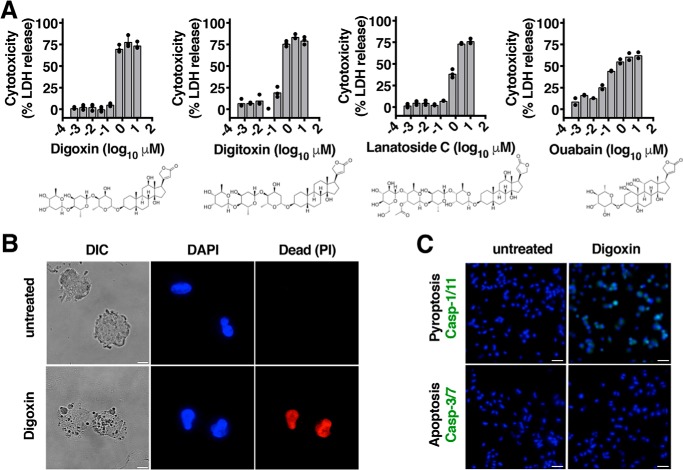
**Cardiac glycosides are cytotoxic.** *A*, death of THP-1 macrophages enumerated by LDH release after 4-h incubation with dilutions of the cardiac glycoside drugs digoxin, digitoxin, lanatoside C, or ouabain. *B*, microscopic examination of THP-1 macrophages treated for 4 h with 1 μm digoxin and stained with DAPI (all cells) and PI (cells with a damaged membrane). Light microscopy (*DIC*) shows disrupted cell architecture at high magnification, with overall morphology consistent with pyroptosis (2). *Scale bar* = 5 μm. *C*, microscopy examination of THP-1 macrophages treated for 4 h with 1 μm digoxin and stained with DAPI (all cells) and FLICA^YVAD^ (caspase-1/11 activity probe, *green*) or FLICA^DEVD^ (caspase-3/7 activity probe, *green*). *Scale bar* = 50 μm. Where applicable, data are represented as mean ± S.D. *n* = 4, representative of at least three independent experiments. Statistical significance was determined by unpaired two-tailed Student's *t* test.

During a routine screen of compounds for cytotoxicity using THP-1 human macrophage-like cells, we found that lanatoside C strongly induced release of lactate dehydrogenase, a canonical indicator of cell lysis (Fig. 1*A*). Cytotoxicity of this drug has been reported previously reported (13), but the rapid induction of death within 2 h and with orders of magnitude greater sensitivity than reported previously suggested that a different mechanistic pathway was causal. Furthermore, we observed that the structurally related cardiac glycosides digoxin, digitoxin, and ouabain exhibited a toxicity similar to lanatoside C (Fig. 1*A*). This also occurred at submicromolar concentrations three orders of magnitude lower than crystalline monosodium urate, a driver of inflammatory pathology in gout and a common positive control for inducing macrophage cell death (17). Cytotoxicity has been reported previously for several cardiac glycosides, with reported IC_50_ values ranging from low nanomolar to high micromolar and the mechanism of cell death varyingly described as apoptosis, necrosis, pyroptosis, or unknown (15, 18). Our observation that these related compounds exhibit a similar toxicity profile suggests that cardiac glycosides likely activate a common cell death pathway.

We next examined digoxin-treated THP-1 macrophages by microscopy for morphological features indicative of a causal cell death mechanism (19). Upon digoxin treatment, cells stained positive with propidium iodide (PI), a membrane-impermanent nuclear dye, and exhibited disrupted membrane integrity, blebbing, and nuclear condensation and leakage (Fig. 1*B*). These are features consistent with caspase-mediated programmed cell death (2, 19). Therefore, we examined activation of the cell death pathways dependent on caspase-3/7 (apoptosis/pyronecrosis) or caspase-1 (pyroptosis) using fluorescent activity-based reporters specific to either caspase family (20). Digoxin treatment activated the FLICA^YVAD^ reporter for caspase-1, the regulator of pyroptosis, but not the FLICA^DEVD^ reporter for apoptotic cell death (Fig. 1*C*).

### Cardiac glycosides activate interleukin-1β

**Figure 2.**
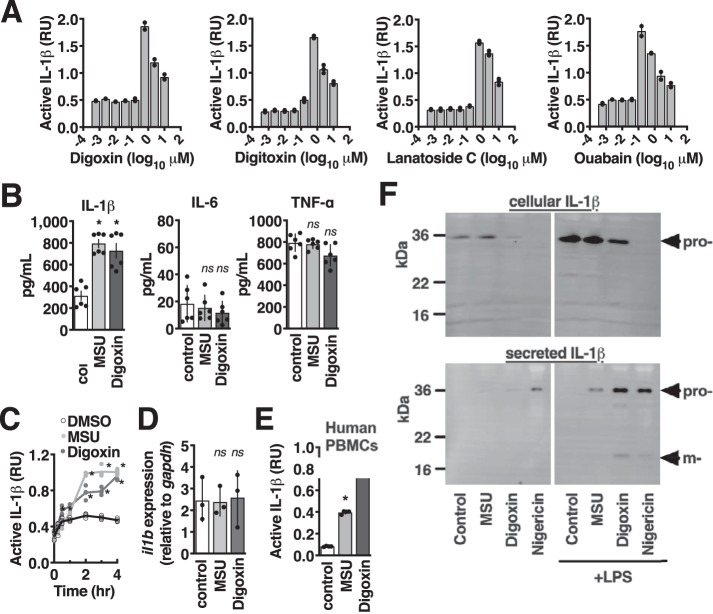
**Cardiac glycosides activate Interleukin-1β.** *A*, IL-1β signaling in supernatant removed from THP-1 macrophages after 4-h incubation with dilutions of the cardiac glycoside drugs digoxin, digitoxin, lanatoside C, or ouabain, assayed using IL-1R bioreporter cells. *RU*, relative units. *B*, THP-1 macrophages incubated with 1 μm digoxin or 1 mm MSU for 4 h and assayed for the proinflammatory cytokines IL-1β, IL-6, and TNFα by ELISA. *C*, kinetics of bioactive IL1β release by THP-1 macrophages treated with 1 μm digoxin or 1 mm MSU. *D*, quantitative RT-PCR of *il1b* transcript levels in THP-1 macrophages treated for 4 h with 1 μm digoxin or 1 mm MSU. *E*, bioactive IL-1β assayed from supernatants of freshly isolated human peripheral blood monocytes treated for 4 h with 1 mm MSU or 100 nm digoxin. *F*, immunoblot examining proteolytic maturation of pro-IL-1β (*pro-*) in cells and supernatent released from THP-1 macrophages (*m-*) with or without 2-h LPS pretreatment and an additional 4-h incubation with 1 mm MSU, 1 μm digoxin, or 20 μm nigericin. Where applicable, data are represented as mean ± S.D. *n* = 4, representative of at least three independent experiments. Statistical significance was determined by unpaired two-tailed Student's *t* test. *, *p* < 0.05; *ns*, not significant.

Caspase-1 regulates not only cell death but also proteolytic maturation and release of the pro-inflammatory cytokine IL-1β (2). To determine whether cardiac glycosides induce this inflammatory signaling, we next used IL-1 receptor–encoding reporter cells as described previously (20). As with cell death, digoxin, digitoxin, lanatoside C, and ouabain were sufficient to induce the release of active IL-1β (Fig. 2*A*). We further confirmed IL-1β release using cytokine-specific enzyme-linked immunosorbent assays. Digoxin treatment resulted in robust secretion of IL-1β and did not induce secretion of IL-6 or TNFα, two other proinflammatory cytokines that are similarly transcriptionally regulated by NF-κB (Fig. 2*B*). Although 2 h of treatment was required for maximal induction of IL-1β signaling, a significant response could be observed as early as 30 min (Fig. 2*C*). One cardiac glycoside, ouabain, was has been reported previously to induce *il1b* transcription (21). However, no difference in *il1b* transcription was detected by quantitative RT-PCR during our experiments (Fig. 2*D*). Like THP-1 cells, peripheral blood monocytes from healthy human donors also released active IL-1β when stimulated with digoxin (Fig. 2*E*).

These rapid activation kinetics further preclude an essentiality of transcriptional regulation and are consistent with digoxin and other cardiac glycosides stimulating rapid posttranslational maturation and release of IL-1β by the inflammasome. To further confirm that the signaling we observed was due to caspase-1 activation, we performed Western blotting on supernatants for IL-1β released upon cell treatment. Treatment with digoxin, MSU, or nigericin increased secretion of IL-1β, concurrent with its cleavage into the 17-kDa mature form by caspase-1 (Fig. 2*E*). When LPS pretreatment of cells was omitted, expression of IL-1β was diminished within the cell, and there was little release of the cytokine in its pro- or mature form. Altogether, these experiments show that cardiac glycosides activate IL-1β through activation of the inflammasome.

### Cardiac glycoside toxicity is evident in human cardiomyocytes

**Figure 3.**
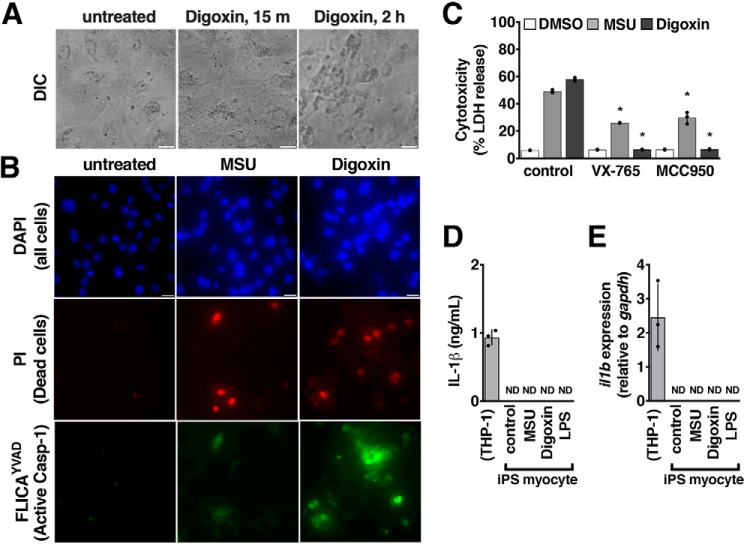
**Cardiac glycoside toxicity is evident in human cardiomyocytes.** *A*, cell imaging of iPS human cardiomyocytes in culture treated with digoxin, demonstrating cell death over time (see Video S1 for contraction). *Scale bars* = 10 μm. *DIC*, differential interference contrast. *B*, microscopy examination of iPS human cardiomyocytes treated for 2 h with 1 μm digoxin or 1 mm MSU and stained with DAPI (all cells), PI (dead cells), and FLICA^YVAD^ (caspase-1/11 activity probe, *green*). *Scale bars* = 10 μm. *C*, death of THP-1 macrophages enumerated by LDH release after 2-h incubation with 1 μm digoxin or 1 mm MSU and 300 nm MCC950 (NLRP3 inhibitor) or 10 μm VX-765 (caspase-1 inhibitor). *D* and *E*, quantification of IL-1β release by ELISA (*D*) and expression by real-time quantitative PCR (*E*) from iPS human cardiomyocytes treated with 1 μm digoxin, 1 mm MSU, or 100 ng/ml LPS compared with THP-1 macrophages. Where applicable, data are represented as mean ± S.D. *n* = 4, representative of at least three independent experiments. *ND*, none detected. Statistical significance was determined by unpaired two-tailed Student's *t* test. *, *p* < 0.05.

To determine whether digoxin directly exerted an inflammatory and cytotoxic effect on cardiac cells, we treated derived human induced pluripotent stem (iPS) cardiomyocytes (22, 23). Upon treatment with 100 nm digoxin, the contractile frequency of iPS cardiomyocytes increased, and synchrony was lost across the culture (Video S1). By 2 h, a larger number of cells were dead, and contractility was further disrupted (Fig. 3*A* and Video S1).

Digoxin-treated iPS cardiomyocytes were permeabilized to PI uptake and stained positive for FLICA^YVAD^, consistent with rapid activation of caspase-1 and cell death observed with THP-1 cells (Fig. 3*B*). As for THP-1 cells and human PBMCs, digoxin induced LDH release from treated cells, and the investigational NLRP3 inflammasome inhibitor MCC950 and the caspase-1 inhibitor VX-765 (24, 25) prevented this lysis (Fig. 3*C*).Unlike with THP-1 cells and human PBMCs, no release of IL-1β could be detected (Fig. 3*D*). We examined the cardiomyocytes by quantitative RT-PCR, and they were confirmed to not express *il1b* (Fig. 3*E*).

### Cardiac glycoside toxicity is evident in human but not murine immune cells

**Figure 4.**
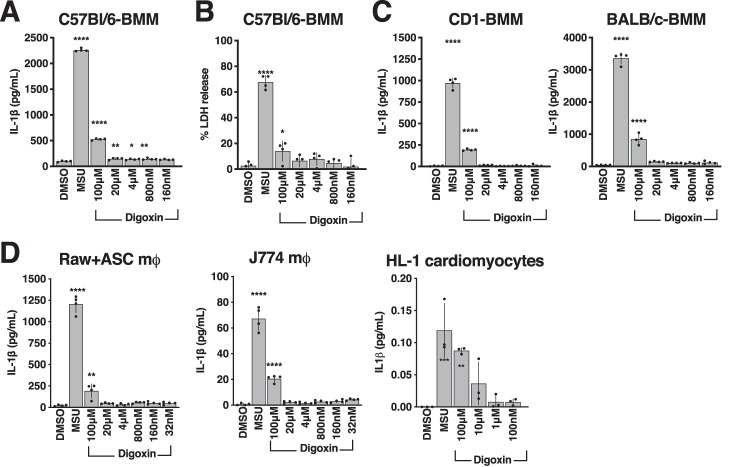
**Cardiac glycoside toxicity is low toward murine cells.** *A* and *B*, BMMs of the C57Bl/6 murine line were incubated for 4 h with dilutions of digoxin or 1 mm MSU, and IL-1β release was quantified by ELISA (*A*), or death of BMMs was quantified by LDH release (*B*). *C*, macrophages derived from the bone marrow of CD1 and BALB/c mice were incubated for 4 h with dilutions of digoxin or 1 mm MSU, and IL-1β release was quantified by ELISA. *D*, macrophages and cardiomyocytes of the indicated murine cell lines were treated for 4 h with dilutions of digoxin or 1 mm MSU, and IL-1β release was quantified by ELISA. Data are represented as mean ± S.D. *n* = 4, representative of at least three independent experiments. Statistical significance was determined by unpaired two-tailed Student's *t* test. *, *p* < 0.05; **, *p* < 0.005; ***, *p* < 0.0005.

We next sought to examine the response to cardiac glycosides using knockout mice deficient in factors key to inflammasome activation of pyroptosis/IL-1β, such as caspase-1, apoptosis-associated specklike protein containing a CARD, the NOD-like receptor, and gasdermin D. However, unlike THP-1 or human primary cells, bone marrow–derived macrophages (BMMs) of the common C57Bl/6 mouse line on which genetic knockout mice are commonly built were unresponsive (as measured by IL-1β release or cytotoxicity) to digoxin except at very high concentrations (>100 μm) (Fig. 4, *A* and *B*). This was specific to cardiac glycosides because the murine cells still released IL-1β in response to the same concentrations of crystalline monosodium urate, used as a positive control (20) (Fig. 4, *A* and *B*). BMMs derived from BALB/c or outbred CD1 mice similarly only responded at very high concentrations (Fig. 4*C*). Additionally, the commonly used murine macrophage cell lines J774 and RAW264.7 and HL-1 murine cardiomyocytes also did not respond to digoxin with the sensitivity observed with human primary cell lines (Fig. 4*D*). Consistent with this finding, 100 μm ouabain has recently been shown to induce NLRP3-dependent IL-1β release in mice and murine cells (14). Together, these results suggest that the lack of cardiac glycoside induction of IL-1β maturation by cells from different sources could be due to the species of origin and not necessarily due to genetic mutations or cell type.

### Pharmacologic targets to ablate cardiac glycoside toxicity

Our observation that three of three human and none of six murine cell types had evidence of inflammasome activation in response to physiologic doses of digoxin suggests that cardiac glycosides activate the inflammasome consequent to their targeting of Na,K-ATPase. The murine homolog was found to be ∼1000 times less sensitive to cardiac glycosides than the human one (16), consistent with a slight IL-1β induction we observed in murine macrophages at high doses (Fig. 4, *A*, *C*, and *D*). Although these concentrations far exceed human toxicity thresholds (26, 27) and target additional processes like T cell differentiation and TNF signaling (14, 28, 29), this finding, mirroring the known differential affinities of digoxin for the human *versus* murine Na,K-ATPase further suggests that the pump could be involved in inflammasome activation.

**Figure 5.**
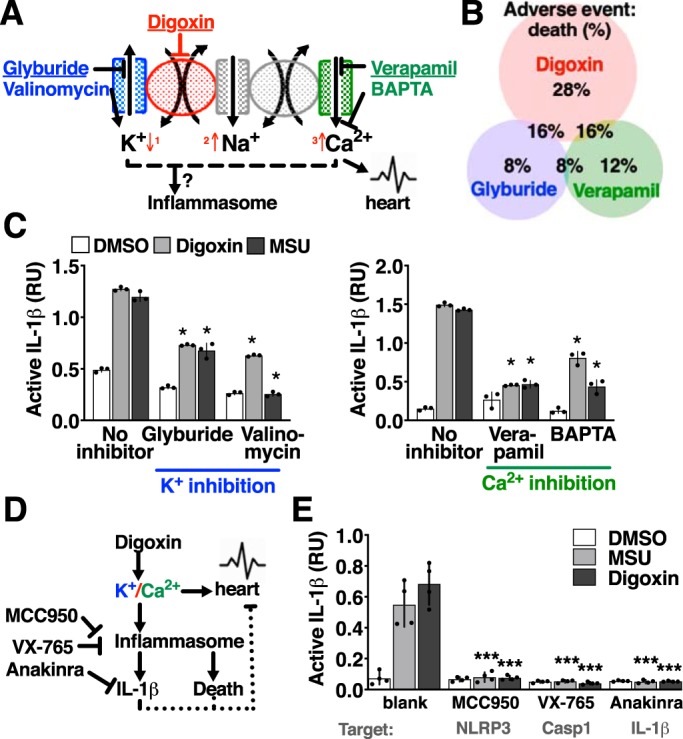
**Pharmacologic targets to ablate cardiac glycoside toxicity.** *A*, model of the digoxin mechanism of action (*red numbers*, sequential ordering of the digoxin effect on intracellular cations) and molecular targets of cation and channel-targeting drugs. *B*, Venn diagram of reports in the FDA Adverse Event Database for the indicated drugs and percentage of those reports resulting in the adverse event of death. *C*, bioactive IL-1β assayed in the supernatant from THP-1 macrophages 4 h after addition of 1 μm digoxin or 1 mm MSU and co-administration of 20 μm glyburide, 10 μm valinomycin, 100 μm verapamil, or 10 μm BAPTA-AM. *RU*, relative units. *D*, model of bifurcation of digoxin effects on the heart and on the inflammasome. *E*, bioactive IL-1β assayed in the supernatant from THP-1 macrophages 4 h after addition of 1 μm digoxin or 1 mm MSU and co-administration of 300 nm MCC950, 10 μm VX-765, or 20 μg/ml anakinra. Where applicable, data are represented as mean ± S.D. *n* = 4, representative of at least three independent experiments. Statistical significance was determined by unpaired two-tailed Student's *t* test. *, *p* < 0.05; ***, *p* < 0.0005.

Cardiac glycosides alter cardiomyocyte contraction by increasing cellular Ca^2+^ consequent to Na,K-ATPase inhibition, which decreases cellular K^+^ (Fig. 5*A*). Many models of inflammasome activation involve cellular flux of K^+^, Ca^2+^, or other markers of cell viability (1, 4, 30–33), potentially integrating both lines of evidence. Another drug that can alter these processes is glyburide, an antidiabetic sulfonylurea that inhibits ATP-sensitive K^+^ channels and can also inhibit inflammasome activation (34). The Ca^2+^ channel blocker verapamil can also inhibit inflammasome activation (35) but can compound the risk for digitalis intoxication by independently slowing the atrioventricular node and impair renal clearance (36, 37). We reviewed post-marketing surveillance from the FDA Adverse Event Reporting System (FAERS) for adverse events occurring in individuals receiving digoxin for any indication. Consistent with the drug's narrow therapeutic range and typical application for congestive heart failure in palliative care, death was a frequent adverse event at 28% of 46,415 reports (Fig. 5*B*). In reports where glyburide or verapamil were present alongside digoxin (1,016 and 1,343 reports, respectively), report of death were fewer, 16% for each combination (Fig. 5*B*). As these epidemiologic associations in post-marketing surveillance suggested that these drugs might alleviate digoxin toxicity, we experimentally tested their potential to inhibit digoxin activation of the inflammasome and secretion of IL-1β. Glyburide and valinomycin, which likewise increase intracellular K^+^, decreased IL-1β release in response to digoxin or MSU (Fig. 4*C*). The calcium channel blocker verapamil and BAPTA-AM, a chelator of intracellular Ca^2+^, also inhibited IL-1β release in response to digoxin or MSU (Fig. 4*C*).

Although targeting of ion transport could potentially alleviate some of the cellular toxicity and inflammation associated with cardiac glycosides, this approach would preclude their therapeutic use. Therefore, we sought to alleviate the inflammation and cell death associated with cardiac glycosides without altering their function. This could be accomplished by more directly targeting the inflammasome and IL-1β (Fig. 5*D*). The investigational NLRP3 inflammasome inhibitor MCC950, the caspase-1 inhibitor VX-765 (24, 25), and the FDA-approved IL-1 receptor antagonist anakinra antagonized IL-1β signaling induced by digoxin (Fig. 5*E*).

## Discussion

Drugs allow human cellular processes to be altered for therapeutic purposes. Prospective drugs are screened for selectivity and deleterious effects, but the diversity and interconnectivity of the body's molecular composition likely make it rare for a pharmacological agent to only interact with a singular, intended target. Inflammasome activation underlies the pathogenesis of numerous inflammatory diseases; therefore, it was with some surprise that we found currently used drugs that activate this response. Furthermore, toxicity occurred at cardiac glycoside concentrations (digoxin EC_50_, ∼100 nm) comparable with those that can be observed in human cardiac muscle (>100 ng/g, ∼128 nm (26, 27)). Because macrophages are robustly sensitive to inflammasome activation in general and in response to cardiac glycosides in particular, we reason that this process could impact cardiac tissue–resident macrophages. These cells directly contribute to electrical activity (38), and the inflammatory factors released by the dying macrophages could influence numerous cellular processes in a paracrine manner (2). Other cells present in the heart muscle will also be exposed to higher cardiac glycoside concentrations than observed in serum, and so would also potentially be subject to direct action by cardiac glycosides and indirect action.

Inflammasome activation is consistent with some of the symptoms of cardiac glycoside intoxication, including fever, increased serum LDH, neutrophilia, inflammatory cell infiltrate, and tissue necrosis (39, 40). These features might be considered secondary to the urgency and severity presented by the cardiac effects of cardiac glycoside intoxication and easily overlooked with the pathology from heart failure. Furthermore, we observed no significant cell death or IL-1β activation at the lower concentrations that that occur in serum, consistent with a previous report that 2 ng/ml digoxin (2.6 nm) did not impact human peripheral blood neutrophil cytotoxicity, chemotaxis, or bactericidal activity (41).

Unseen benefits from inhibiting IL-1β or the inflammasome may already be lowering the incidence of serious adverse events from cardiac glycosides in patients incidentally taking anti-inflammatories for other indications. In a natural experiment examining this possibility, the FAERS contained only 10 reports with the co-incidence of anakinra and digoxin, of which there were two deaths, one because of osteonecrosis and one because of cardiac failure. Thus, although our *in vitro* results suggest a mechanism and the possibility of a therapeutic benefit for IL-1β/inflammasome inhibition during cardiac glycoside treatment, there is insufficient evidence that this has previously occurred in a human. Nonetheless, this strategy may provide a supportive therapeutic option for intoxication alongside conventional pacing and tachycardia with magnesium (42) and in cases when treatment with neutralizing antibodies (43) is not an option, such as during intoxication with naturally occurring cardiac glycosides. Anakinra and inflammasome-targeting drugs are increasingly being investigated for other indications relevant to heart failure (44), myocardial infarction (45), and frequent co-morbidities such as atherosclerosis (46), chronic obstructive pulmonary disease (47), type II diabetes (48, 49), and obesity (50). Such drug–drug interactions are likely to increase in frequency as the therapeutic value of IL-1 inhibition is explored, so protective synergies may yet be found during the future course of clinical studies.

## Materials and methods

### Cell culture

THP-1 human monocytes (ATCC, TIB-202), RAW-ASC (ATCC, TIB-71 transformed with pcDNA3-Myc-ASC (Addgene plasmid 73952 (51))), and J774 murine macrophages (ATCC, TIB-67) were cultured in RPMI medium supplemented with 0.05 mm 2-mercaptoethanol, 0.2% D-glucose, 10 mm HEPES, 1 mm sodium pyruvate, 10% fetal bovine serum, and 10,000 units/ml penicillin and streptomycin. Where indicated, THP-1 cells were differentiated into macrophage-like cells with 72-h incubation with 200 nm phorbol 12-myristate 13-acetate. All cells were primed, except where indicated, with lipopolysaccharide (LPS, Sigma) to 100 ng/ml 2 h before the experiment (52).

Primary macrophages were generated from femur exudates of C57Bl/6 (The Jackson Laboratory), CD1 (The Jackson Laboratory), or BALB/c (The Jackson Laboratory) mice as described previously (20). Human peripheral blood monocytes (PBMCs) were isolated by density graduation (Accurate Chemical and Scientific Corp.) from whole blood of healthy volunteers after informed consent abiding by the Declaration of Helsinki principles and as approved by the UCSD Institutional Review Board/Human Research Protection Program under protocol 070278X.

iPS cell–derived cardiomyocytes were provided by Cellular Dynamics or prepared in the laboratory as described previously (23). HL-1 cardiomyocytes were cultured in Claycomb media (53). Cardiomyocytes were plated on 96-well plates prepared with a thin coating of Matrigel (protein concentration, 100 μg/ml) at ∼125,000 cells/cm^2^.

Digoxin (Sigma-Aldrich or Hikma Farmaceutica), digitoxin (Sigma-Aldrich), ouabain (Sigma-Aldrich), lanatoside C (Sigma-Aldrich), monosodium urate (MSU) crystals (Invivogen), 1,2-bis[2-aminophenoxy] ethane-*N*,*N*,*N*0,*N*0-tetraacetic acid-acetoxymethyl ester (BAPTA-AM, Calbiochem), glyburide (glybencamide, Sigma-Aldrich), valinomycin (Sigma-Aldrich), verapamil (Sigma-Aldrich), anakinra (Amgen), VX-765 (Invivogen), and MCC950 (Invivogen) were diluted in PBS and incubated with cells at the indicated concentrations for 2 h unless otherwise noted.

### Cytotoxicity measurements

Cell death was quantified by release of lactate dehydrogenase from cells plated at 20,000 cells/well in a 96-well plate format following the manufacturer's protocol (Cytotox 96 kit, Promega). The percentage of cell death was calculated after subtraction of untreated control cells and division by a positive control of cells treated with lysis solution.

Caspase activation was determined by FLICA^YVAD^ (FAM-YVAD-fmk, caspase-1/11) and FLICA^DEVD^ (FAM-DEVD-fmk, caspase-3/7) according to the manufacturer's protocol (Immunochemistry Technologies) with parallel cell permeability monitoring with 10 μg/ml propidium iodide/DAPI (Immunochemistry Technologies) as described previously (52). Cells were seeded at 200,000 cells/ml on coverslips and observed by wide-field fluorescence microscopy using ×63 or ×20 objectives (Axio Observer D1 or Axio Observer Z1, Carl Zeiss).

### Cytokine measurements

Relative IL-1 signaling was measured by removing 50 μl of supernatant from treated cells (*i.e.* macrophages or cardiomyocytes) onto transgenic IL-1β reporter cells (Invivogen), and secreted alkaline phosphatase activity was measured after 18 h of co-incubation as described previously (20). IL-1β, IL-6, and TNFα were quantified by ELISA (R&D Systems). Cells were lysed with radioimmune precipitation assay buffer (Millipore), RNA was isolated (Qiagen), complementary DNA was synthesized with SuperScript III and Oligo(dT)_20_ primers (Invitrogen), and quantitative PCR was performed with KAPA SYBR Fast (Kapa Biosystems) and primers for *il1b* (ATGATGGCTTATTACAGTGGCAA and GTCGGAGATTCGTAGCTGGA) and *gapdh* (TGTGGGCATCAATGGATTTGG and ACACCATGTATTCCGGGTCAAT), with normalization to *gapdh*. Relative expression compared using the ΔΔCt method.

Western blots for IL-1β cleavage were performed directly on cell lysates or cell supernatants concentrated using a 3-kDa centrifugal filter (Millipore). Equal volumes were separated on a Tris/glycine gel, and proteins were transferred to PVDF. Blots were probed with anti-IL-1β antibody (R&D Systems) followed by DyLight 650 secondary antibody (Novus Biologicals) and imaged with a BioRad ChemiDoc MP instrument.

### Statistical analysis

Statistical significance was calculated by unpaired Student's *t* test (*, *p* < 0.05; **, *p* < 0.005; ***, *p* < 0.0005) using GraphPad Prism unless otherwise indicated. Data are representative of at least three independent experiments. Input data for adverse event analysis were taken from the public release of the FDA's FAERS database, covering the period from 2000 through 2017. Each drug indicated was queried individually or in the combinations indicated, and the total number of records matching the terms was recorded. Each query was re-run with death as the reported outcome to provide the percentage of mortality of the indicated reports in the database.

## Author contributions

D. L. L., V. N., and C. N. L. conceptualization; D. L. L., V. N., and C. N. L. resources; D. L. L., J. S. S., V. N., and C. N. L. data curation; D. L. L., V. N., and C. N. L. formal analysis; D. L. L., V. N., and C. N. L. supervision; D. L. L. and C. N. L. validation; D. L. L., J. S. S., E. E., M. R., and C. N. L. investigation; D. L. L. and C. N. L. visualization; D. L. L., P. J. B., E. D. A., V. N., and C. N. L. methodology; D. L. L. and C. N. L. writing-original draft; D. L. L., V. N., and C. N. L. project administration; D. L. L., V. N., and C. N. L. writing-review and editing; V. N. and C. N. L. funding acquisition.

## Supplementary Material

Supporting Information

## Footnotes

3 The abbreviations used are:

FDA: Food and Drug Administration
PI: propidium iodide
TNF: tumor necrosis factor
MSU: monosodium urate
iPS: induced pluripotent stem
PBMC: peripheral blood monocyte
BMM: bone marrow-derived macrophage
FAERS: FDA Adverse Event Reporting System
BAPTA-AM: 1,2-bis[2-aminophenoxy] ethane-*N*,*N*,*N*0,*N*0-tetraacetic acid-acetoxymethyl ester
LDH: lactate dehydrogenase
LPS: lipopolysaccharide
DAPI: 4′,6-diamidino-2-phenylindole
ASC: apoptosis-associated specklike protein containing a CARD
NLR: NOD-like receptor.
