# Supplementary material for: Inflammasome inhibition blocks cardiac glycoside cell toxicity
Source: J Biol Chem. 2019 Jul 12;294(34):12846–54. doi: 10.1074/jbc.RA119.008330 (PMC6709640; doi:10.1074/jbc.RA119.008330)
Supplement: Supporting Information [file supp_294_34_12846__index.html]

Inflammasome inhibition blocks cardiac glycoside cell toxicity — Inflammasome activation by cardiac glycosides — Inflammasome inhibition blocks cardiac glycoside cell toxicity — Inflammasome activation by cardiac glycosides — Supporting Information 

# Inflammasome inhibition blocks cardiac glycoside cell toxicity

## Supporting Information

- Supporting Information (to be published online) - Video S1, related to Fig 3a
